# Hypomethylation‐Enhanced F‐Box Protein 32 Promotes Hepatocellular Carcinogenesis via Ubiquitin‐Mediated PHLPP2 Degradation

**DOI:** 10.1002/mco2.70410

**Published:** 2025-09-23

**Authors:** Shu Chen, Kai Yu, Zhengming Deng, Xiaopei Hao, Ping Shi, Zhengzheng Wang, Jiali Xu, Jingjing Dai

**Affiliations:** ^1^ Department of Hepatobiliary Pancreatic Spleen Surgery The Affiliated Hospital of Jiangsu University Zhenjiang China; ^2^ Hepatobiliary Center, The First Affiliated Hospital With Nanjing Medical University; Key Laboratory of Liver Transplantation, Chinese Academy of Medical Sciences; NHC Key Laboratory of Hepatobiliary Cancers Nanjing Jiangsu Province China; ^3^ Department of General Surgery, Jiangsu Province Hospital of Chinese Medicine Affiliated Hospital of Nanjing University of Chinese Medicine Nanjing China; ^4^ Department of Hepatobiliopancreatic Surgery The Affiliated Cancer Hospital of Zhengzhou University & Henan Cancer Hospital Zhengzhou China; ^5^ Department of Infectious Diseases The First Affiliated Hospital With Nanjing Medical University Nanjing Jiangsu Province China; ^6^ Department of Anesthesiology, Jinling Hospital, Affiliated Hospital of Medical School Nanjing University Nanjing Jiangsu Province China

**Keywords:** biomarker, DNA methylation, E3 ubiquitin ligase, PI3K–AKT pathway

## Abstract

F‐Box Protein 32 (FBXO32), a F‐box protein family member, exhibits oncogenic and tumor‐suppressive roles in various carcinomas. However, its function and underlying molecular mechanisms in hepatocellular carcinoma (HCC) are still unknown. We observed that FBXO32 was overexpressed in HCC tissues than normal tissues, which is pertaining to poor prognosis in HCC patients. Functional tests demonstrated that FBXO32 enhanced HCC cell proliferation, invasion, and metastasis, which was confirmed in vivo using mouse models. Proteomics‐based approaches and computational analyses reported a positive correlation between FBXO32 and PI3K–AKT pathway, identifying pleckstrin homology domain leucine‐rich repeat protein phosphatase 2 (PHLPP2) as an interacting protein. Mechanistically, DNA promoter hypomethylation elevated FBXO32 expression in HCC cells, promoting K48‐linked PHLPP2 polyubiquitination at the K592 and K942 sites through direct interactions. Notably, targeting FBXO32 significantly inhibited tumor growth in both an orthotopic HCC model and an organoid model derived from HCC patients. To sum up, this work emphasizes the part of FBXO32 in propelling HCC progression via facilitating PI3K–AKT pathway activation via PHLPP2 degradation.

## Graphical abstract

F‐Box Protein 32 (FBXO32) is markedly overexpressed in hepatocellular carcinoma and facilitates tumor progression by activating the PI3K–AKT signaling pathway via PHLPP2 degradation, underscoring its potential as a promising therapeutic target.

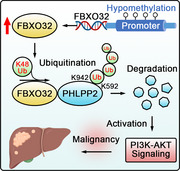

## 1 Introduction

Hepatocellular carcinoma (HCC) is a common malignancy and a leading cause of cancer‐related deaths globally [1]. Despite progress in diagnosis and treatment, the overall HCC prognosis remains poor, primarily owing to its complex pathological mechanisms and high heterogeneity [2]. The late‐stage diagnosis, limited treatment options, and frequent resistance to conventional therapies contribute to the poor survival rates of HCC patients [3]. Hence, clearly understanding molecular mechanisms driving HCC development and advance is urgently needed to recognize novel therapeutic targets.

Recently, researchers have increasingly recognized critical role of ubiquitin–proteasome system (UPS) in various cancers’ occurrence and development [4, 5, 6, 7]. The UPS is the primary pathway for intracellular protein modification through ubiquitin‐mediated substrate alteration [8, 9]. The UPS regulates various cellular processes, like signal transduction, protein degradation, transportation, cell cycle, and apoptosis, making it a central regulator of cellular homeostasis. UPS dysregulation has been extensively reported in HCC involving tumor malignancy, immune evasion, and angiogenesis [10, 11, 12].

The UPS involves coordinated action of three enzymes: ubiquitin‐activating enzyme (E1), ubiquitin‐conjugating enzyme (E2), and ubiquitin ligase (E3) [13]. Among these, E3 ligases are critical to measuring ubiquitination substrate specificity and selectivity. Variations in E3 ligase expression or activity are critical in cancer and influence tumor progression, development, and drug response [14, 15]. E3 ligases exhibit oncogenic or tumor‐suppressive properties via adjusting different substrates’ ubiquitination [16, 17]. Therefore, targeting specific E3 ligases has become alternative cancer therapy.

As a significant component of E3 ligases, the F‐box protein family participates in multiple cancer‐related signaling pathways through the ubiquitination and degradation of targeted substrates [18]. Their potential applications in cancer diagnosis and therapy have garnered extensive attention in preclinical studies [19]. F‐Box Protein 32 (FBXO32), in particular, is crucial to malignant tumor progression [20, 21]. FBXO32 ubiquitinates eEF1A1, thereby stimulating protein synthesis and driving pancreatic cancer progression and metastasis [22]. FBXO32 regulates ubiquitination and nuclear translocation of CtBP1, promoting epithelial–mesenchymal transition (EMT) [23]. Additionally, FBXO32 facilitates lung adenocarcinoma migration and invasion by ubiquitinating and degrading PTEN [24]. However, despite the established significance of F‐box proteins in various cancer types, their specific role and clinical value in HCC need more survey.

In our work, we found that DNA promoter hypomethylation elevated FBXO32 expression in HCC cells. Upregulated FBXO32 promotes ubiquitin–proteasome degradation of pleckstrin homology domain leucine‐rich repeat protein phosphatase 2 (PHLPP2) through direct interactions, accordingly activating PI3K–AKT pathway and subsequently promoting HCC progression. To sum up, our work exhibits that FBXO32 is crucial to HCC occurrence and development and probably act as a potential HCC treatment target.

## 2 Results

### 2.1 FBXO32 Overexpression in HCC Correlates With Poor Prognosis

**FIGURE 1:**
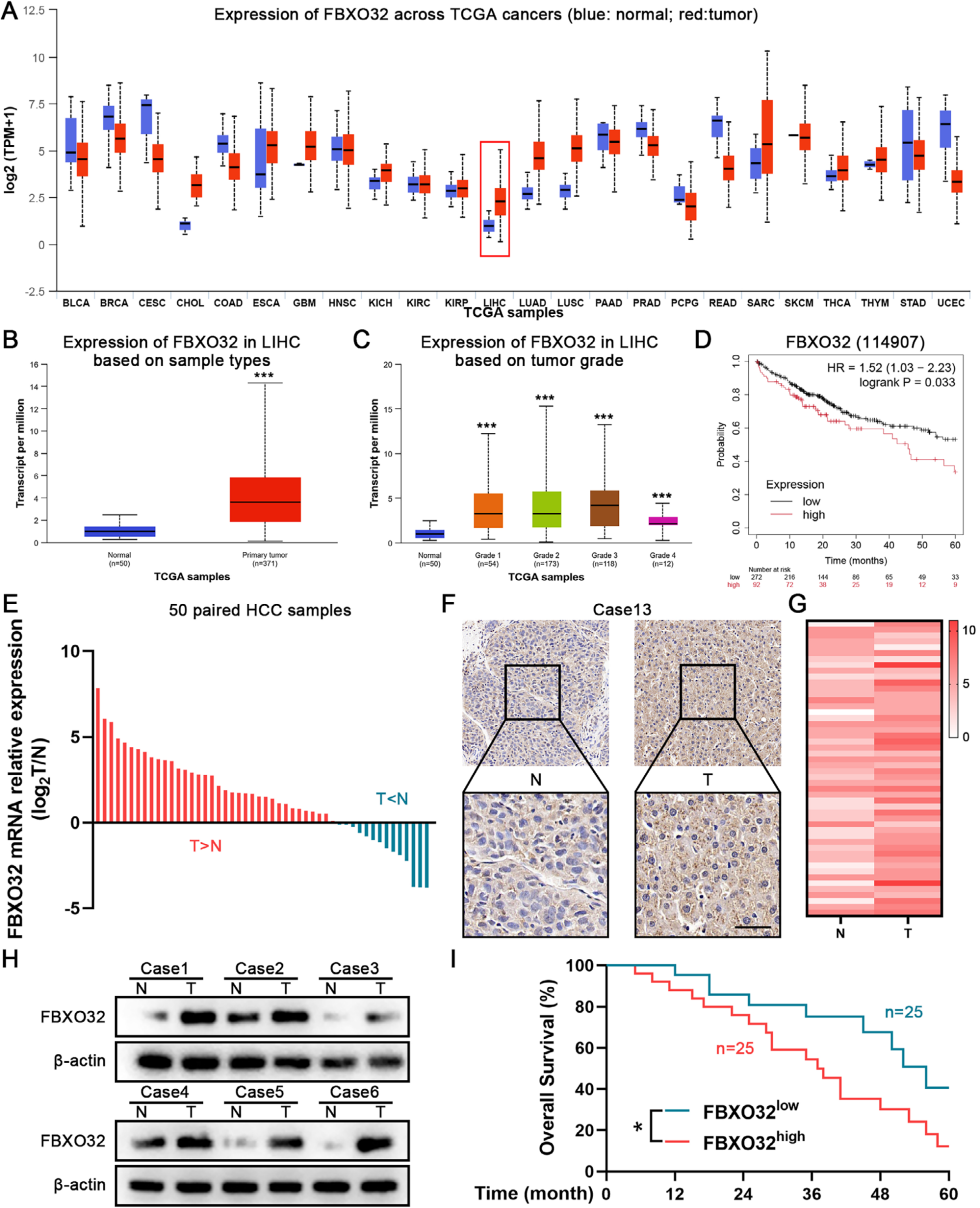
F‐Box Protein 32 (FBXO32) is overexpressed in HCC and correlated with poor prognosis. (A) Dot plot comparing the *FBXO32* mRNA expression levels in tumor and normal tissues from the TCGA database. (B) Dot plot comparing the *FBXO32* mRNA expression levels in cancerous and normal liver tissues from the TCGA database. (C) Upregulated *FBXO32* mRNA expression was significantly associated with patient tumor grade and stage in HCC. (D) Kaplan–Meier analysis showing the overall survival of patients with diverse FBXO32 expression. (E) Waterfall plot showing *FBXO32* mRNA expression fold‐change in 50 pairs of HCC and adjacent nontumorous tissues in log scale. (F, G) Representative images of FBXO32 expression in paired HCC specimens and immunohistochemical (IHC) analysis. *n* = 50 per group; scale bars: 50 mm. (H) FBXO32 protein expression detected in six paired HCC and matched adjacent normal tissues. (I) Survival analysis using the Kaplan–Meier method after categorizing HCC patients into low or high FBXO32 expression groups, *n* = 25 per group. All data are presented as the means ± SD, *p < 0.05; **p < 0.01; ***p < 0.001; ns, not significant.

For investigating FBXO32's part in HCC, we exploited RNA‐Seq data from Cancer Genome Atlas (TCGA) database to obtain *FBXO32* expression profiles in normal and cancer tissues. Our outcomes indicated that FBXO32 was significantly overexpressed in liver cancer cells (Figure 1A,B). FBXO32 expression was also progressively elevated in advanced stages of liver cancer (Figure 1C). Additionally, FBXO32 overexpression was significantly pertaining to poor overall survival (OS) in liver cancer patients (Figure 1D). Such outcomes indicate that FBXO32 might be crucial to HCC. Therefore, we focused our research on FBXO32.

To validate our bioinformatic analysis, we analyzed 50 pairs of HCC and corresponding adjacent nontumorous liver tissue specimens using qRT‐PCR. *FBXO32* mRNA expression notably upregulated in HCC tissues than in adjacent tissues (Figure 1E). Immunohistochemical (IHC) analysis revealed significantly higher FBXO32 staining scores in tumor tissues (Figure 1F,G). We chose six pairs of HCC samples at random to survey FBXO32 protein expression and found that FBXO32 protein levels were remarkably risen in HCC tissues than in matched adjacent normal tissues (Figure 1H). Moreover, survival analysis implied a correlation between increased FBXO32 expression and poor OS in HCC patients (Figure 1I).

### 2.2 FBXO32 Promotes HCC Cell Proliferation

**FIGURE 2:**
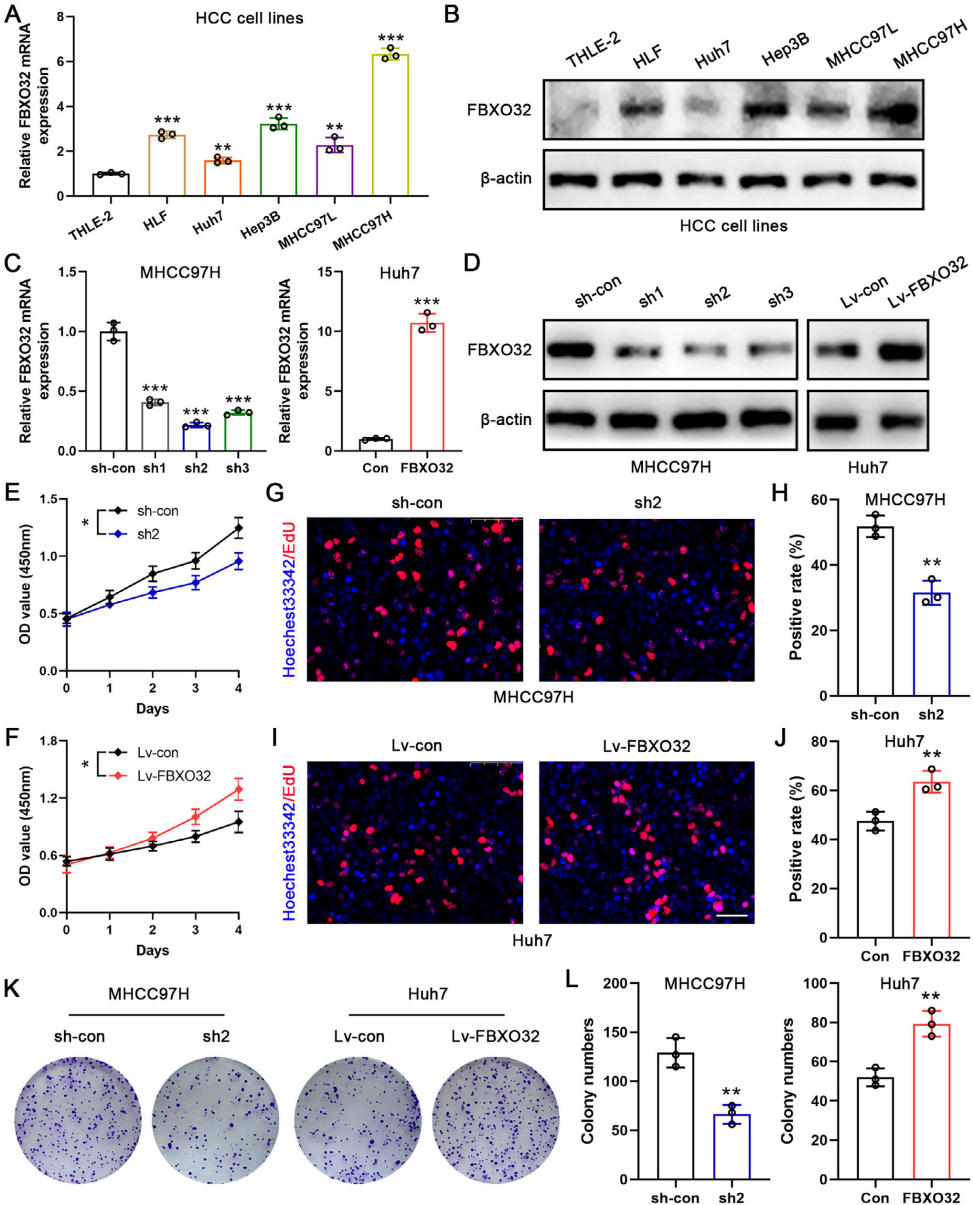
FBXO32 promotes HCC cell proliferation. (A, B) FBXO32 mRNA and protein expression levels in normal liver cell line (THLE‐2) and HCC cell lines (HLF/Huh7/Hep3B/MHCC97L/MHCC97H) as measured by qRT‐PCR and western blotting analysis, respectively (*n* = 3). (C, D) FBXO32 mRNA and protein expression in MHCC97H and Huh7 cells. β‐Actin served as loading control (*n* = 3). (E, F) CCK8 results of MHCC97H and Huh7 cells (*n* = 3). (G, H) EdU assay results and statistics of the proliferation efficiency of MHCC97H cells in the indicated groups (*n* = 3). Scale bar, 50 µm. (I, J) EdU assay results and statistics on the proliferation efficiency of Huh7 cells in the indicated groups (*n* = 3). Scale bar, 50 µm. (K, L) Colony formation and its statistical results for MHCC97H and Huh7 cells in the indicated groups (*n* = 3). All data are presented as the means ± SD, *p < 0.05; **p < 0.01; ***p < 0.001; ns, not significant.

Next, we measured FBXO32 expression levels in a normal liver cell line (THLE‐2) and various HCC cell lines (HLF, Huh7, Hep3B, MHCC97L, and MHCC97H) by qRT‐PCR and western blotting. We found that FBXO32 mRNA and protein expression in HCC cells, particularly MHCC97H cells, was higher than those in THLE‐2 cells (Figure 2A,B). For keeping probing into FBXO32's role in HCC, we transfected MHCC97H cells utilizing short hairpin RNA (shRNAs) targeting FBXO32 (sh1, sh2, and sh3), whereas Huh7 cells with low FBXO32 expression were infected with a lentivirus to overexpress FBXO32 (Lv‐FBXO32). Transfection efficiency was validated in each group, with sh2 demonstrating significant MHCC97H cell inhibition. Additionally, Lv‐FBXO32 successfully induced FBXO32 overexpression in Huh7 cells, and both were subsequently used for functional experiments (Figure 2C,D).

Cell Counting Kit‐8 (CCK‐8) assay outcomes indicated that FBXO32 knockdown suppressed MHCC97H cell viability, whereas FBXO32 overexpression significantly increased Huh7 cell viability (Figure 2E,F). Furthermore, EdU assays verified that FBXO32 overexpression and knockdown in Huh7 cells resulted in more and fewer EdU‐positive cells, respectively, than in control cells (Figure 2G–J). Additionally, plate clone formation assays exhibited that low FBXO32 expression dramatically restrained MHCC97H cell proliferation, while FBXO32 overexpression enhanced Huh7 cell proliferation (Figure 2K,L).

### 2.3 FBXO32 Promotes HCC Malignant Phenotypes

**FIGURE 3:**
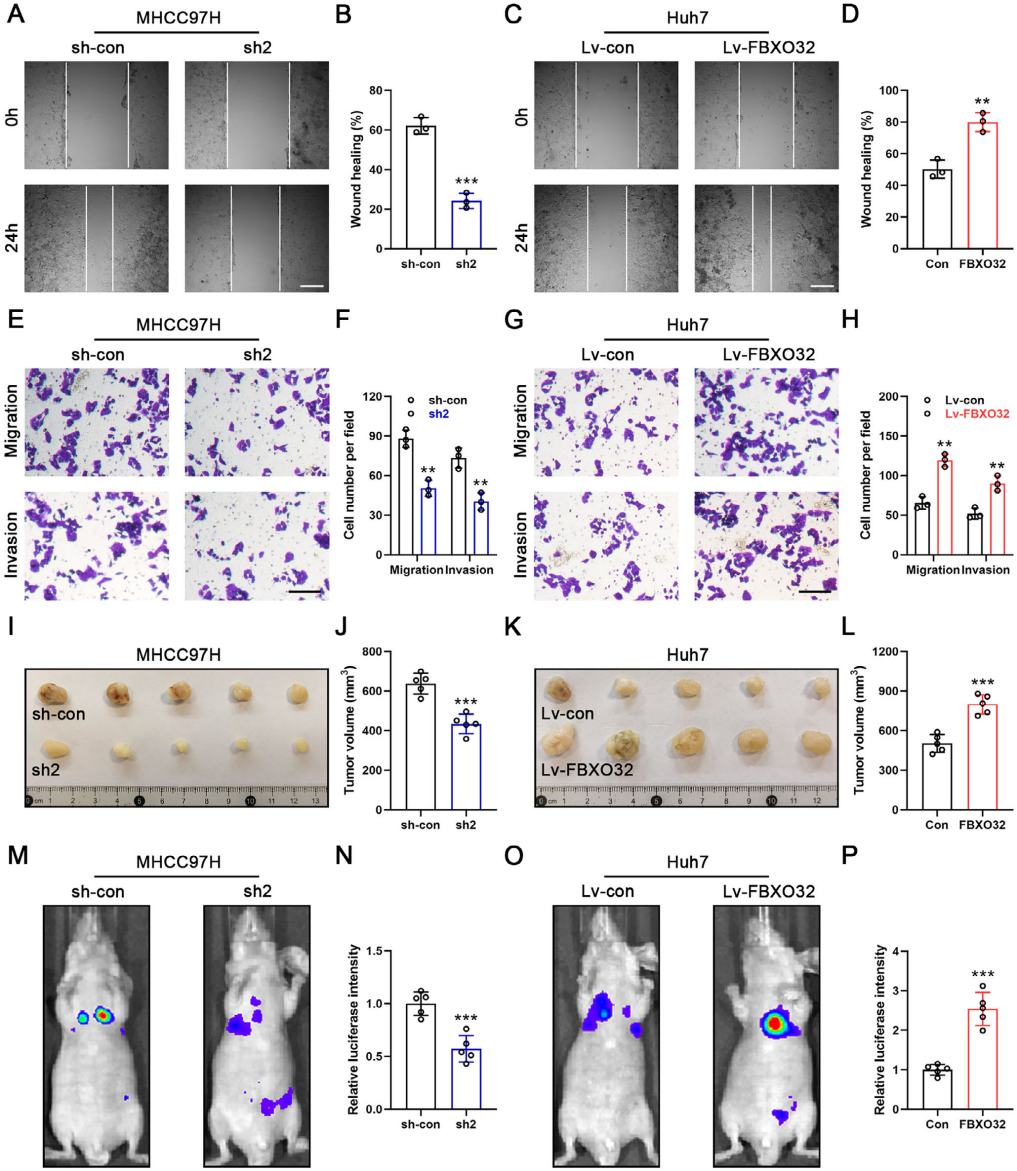
FBXO32 regulates HCC malignant phenotypes. (A, B) The percentage of wound width at 0 and 24 h postwound formation in MHCC97H cells (*n* = 3). Scale bar, 200 µm. (C, D) The percentage of wound width at 0 and 24 h postwound formation in Huh7 cells (*n* = 3). Scale bar, 200 µm. (E, F) Transwell assays of MHCC97H cells after FBXO32 knockdown. Migrating and invading cells per field were counted and analyzed (*n* = 3). Scale bar, 50 µm. (G, H) Transwell assays of Huh7 cells after FBXO32 overexpression. Migrating and invading cells per field were counted and analyzed (*n* = 3). Scale bar, 50 µm. (I, J) Representative images of xenograft tumors formed by subcutaneous injection of MHCC97H‐transfected cells (*n* = 5). Statistical analysis of xenograft tumor volume. (K, L) Representative images of xenograft tumors formed by subcutaneous injection of Huh7‐transfected cells (*n* = 5). Statistical analysis of xenograft tumor volume. (M, N) Representative images of in vivo luminescence captured after intravenous injection of MHCC97H‐transfected cells (*n* = 5). Statistical analysis of luciferase intensity. (O, P) Representative images of in vivo luminescence captured after intravenous injection of Huh7‐transfected cells (*n* = 5). Statistical analysis of luciferase intensity. All data are presented as the means ± SD, *p < 0.05; **p < 0.01; ***p < 0.001; ns, not significant.

Wound healing and transwell assays showed that FBXO32 knockdown significantly inhibited HCC cell migration and invasion. Opposite effects were observed with FBXO32 overexpression, which significantly enhanced the malignant phenotype (Figure 3A–H). To explore FBXO32's impact upon tumor growth in vivo, we created a mouse subcutaneous xenograft model. We subcutaneously injected FBXO32‐knockdown MHCC97H cells or FBXO32‐overexpressing Huh7 cells, along with the corresponding control HCC cells, into nude mice. Tumor size decreased in the FBXO32 knockdown group and increased in the FBXO32 overexpression group (Figure 3I–L). To further survey FBXO32 on HCC metastasis in vivo, we created a lung metastasis model via intravenously injecting luciferase‐positive HCC cells into nude mice. After 3 weeks, the FBXO32 knockdown group exhibited a significantly decreased fluorescence intensity than the control group, indicating reduced metastasis. In contrast, FBXO32 overexpression promoted cell metastasis (Figure 3M–P). In summary, our findings confirm that FBXO32 promotes malignant phenotypes of HCC both in vitro and in vivo.

### 2.4 FBXO32 Promotes HCC by Enhancing AKT Phosphorylation

**FIGURE 4:**
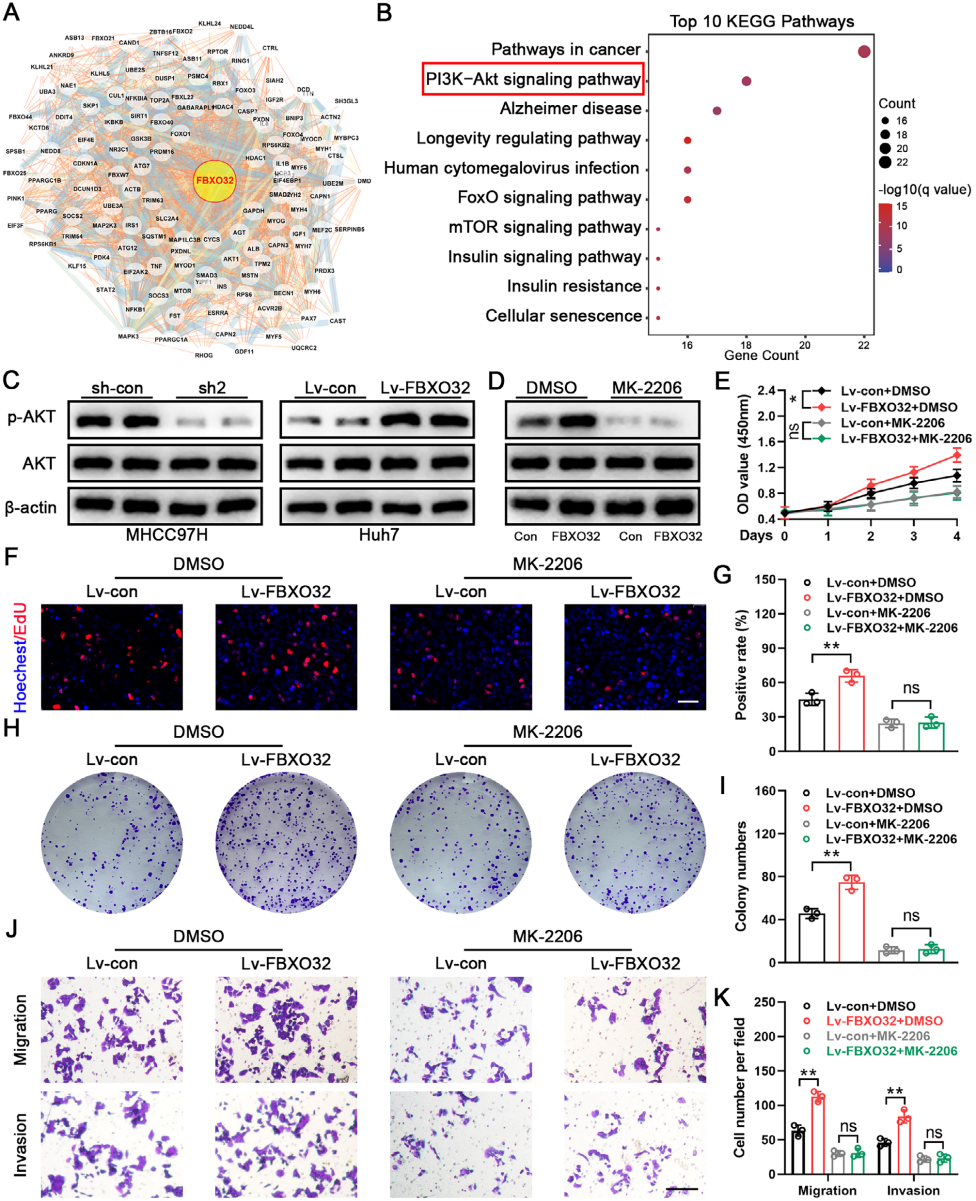
FBXO32 regulates the AKT signaling pathway. (A) The protein interaction network of FBXO32 analyzed using the STRING database. (B) Gene set enrichment plots showing FBXO32 association with the PI3K–AKT signaling pathway. (C) Total and phosphorylated AKT levels in MHCC97H and Huh7 cells, with β‐actin serving as the loading control. (D) Western blotting analysis of Lv‐con and Lv‐FBXO32 Huh7 cells treated with 1 µM MK‐2206 or DMSO. (E) CCK‐8 analysis of Lv‐con and Lv‐FBXO32 Huh7 cells treated with MK‐2206 and DMSO (*n* = 3). (F, G) EdU assay results for Lv‐con and Lv‐FBXO32 Huh7 cells treated with MK‐2206 and DMSO (*n* = 3). Scale bar: 50 µm. (H, I) Representative colony formation images and their statistical analysis for Lv‐con and Lv‐FBXO32 Huh7 cells treated with MK‐2206 or DMSO (*n* = 3). (J, K) Transwell assay images for migration and invasion, along with their statistical analysis for Lv‐con and Lv‐FBXO32 Huh7 cells treated with MK‐2206 and DMSO (*n* = 3). Scale bar: 50 µm. All data are presented as the means ± SD, *p < 0.05; **p < 0.01; ***p < 0.001; ns, not significant.

We elucidated FBXO32's part in propelling HCC cell proliferation, invasion and migration in vitro and demonstrated that FBXO32 enhanced HCC growth and migration in vivo. However, precise mechanism by which FBXO32 exerts these effects remains unknown. For addressing this, we analyzed the FBXO32 protein interaction network utilizing STRING database (Figure 4A) and conducted Kyoto Encyclopedia of Genes and Genomes (KEGG) pathway enrichment analysis. Our findings implied that FBXO32‐related proteins are involved in various cancer‐associated pathways, particularly PI3K–AKT signaling pathway (Figure 4B).

For validating if FBXO32 activates this pathway in HCC, we determined pathway‐related proteins’ expression via western blotting. Our findings indicated that phosphorylated AKT (p‐AKT) level decreased in FBXO32‐knockdown MHCC97H cells, but increased in FBXO32‐overexpressing Huh7 cells (Figure 4C). For survey if AKT activity mediates FBXO32 function in HCC progression, we treated Huh7 cells utilizing p‐AKT inhibitor, MK‐2206 (Figure 4D). CCK‐8, EdU, and colony formation assays implied that MK‐2206 abolished the promotive effects of FBXO32 overexpression upon Huh7 cell viability and proliferation (Figure 4E–I). Moreover, MK‐2206 restrained migration and invasion reinforced via FBXO32 overexpression (Figure 4J,K). Collectively, such outcomes demonstrated that FBXO32 activates AKT signaling pathway to promote HCC progression.

### 2.5 FBXO32 Promotes PHLPP2 Ubiquitination and Proteasomal Degradation

**FIGURE 5:**
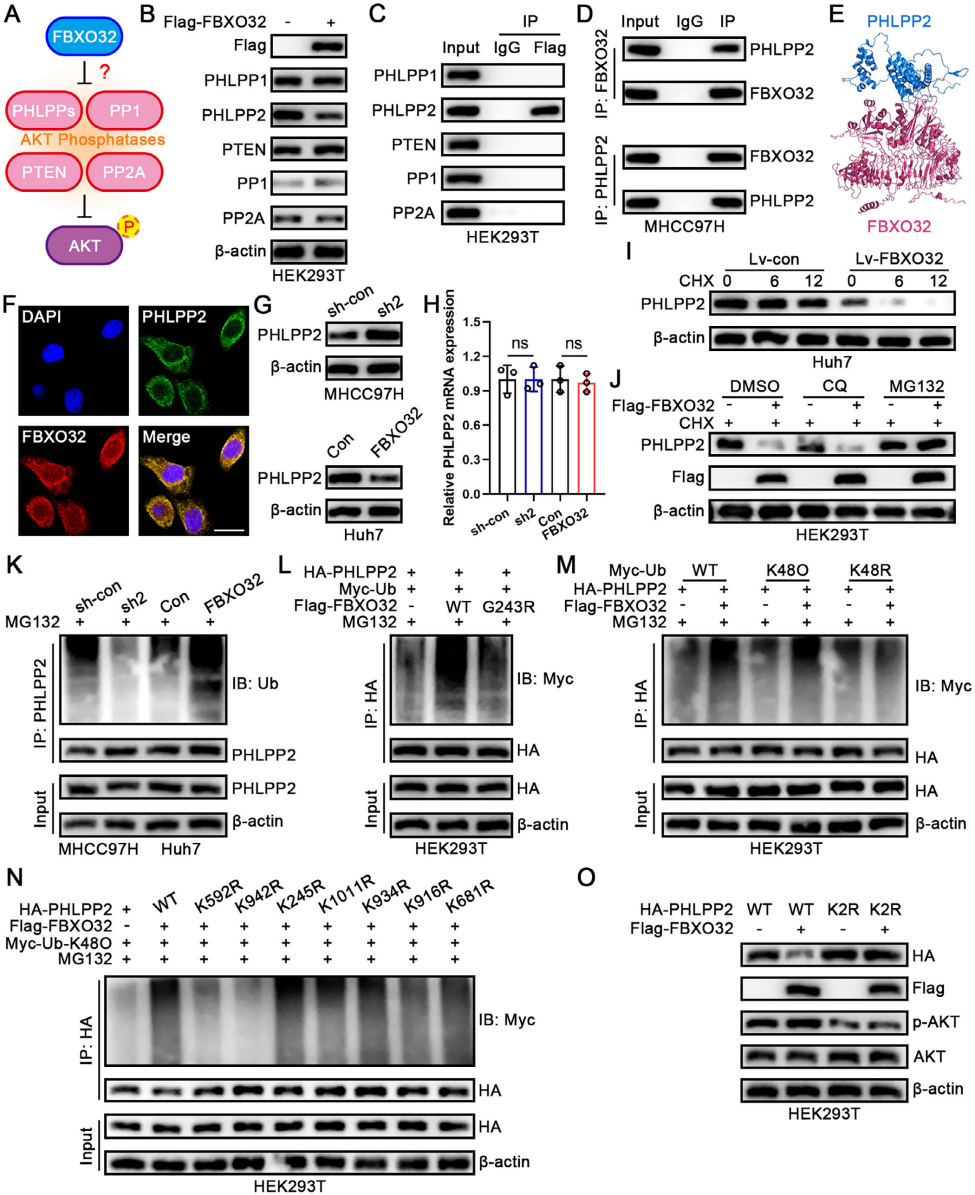
FBXO32 promotes PHLPP2 ubiquitination and proteasomal degradation. (A) Schematic representation illustrating how FBXO32 induces HCC progression via the PI3K–AKT pathway. (B) Western blotting analysis of FBXO32‐overexpressing HEK293T cells with the indicated antibodies. (C) Immunoprecipitation (IP) assay confirming candidate‐binding FBXO32 proteins in HEK293T cells. (D) Endogenous IP assay confirming the binding of FBXO32 with PHLPP2 in MHCC97H cells. (E) The potential binding conformation of FBXO32 with PHLPP2 predicted using the HDOCK SERVER, resulting in a docking score of −258.60. (F) Immunofluorescence image of FBXO32 and PHLPP2 colocalization. Scale bar, 10 µm. (G) PHLPP2 protein expression levels after FBXO32 knockdown or overexpression in MHCC97H and Huh7 cells. (H) *PHLPP2* mRNA expression levels after FBXO32 knockdown or overexpression in MHCC97H and Huh7 cells (*n* = 3). (I) Western blotting analysis of PHLPP2 protein levels in Lv‐FBXO32‐transfected Huh7 cells treated with 50 µg/mL cycloheximide (CHX), a protein synthesis inhibitor, for the indicated time points. (J) PHLPP2 expression in FBXO32‐overexpressing HEK293T cells treated with 50 µg/mL CHX in combination with 10 µM MG132, a proteasome inhibitor, or 20 µM chloroquine (CQ), a lysosome inhibitor. DMSO treatment was used as a control. (K) PHLPP2 ubiquitination after FBXO32 knockdown or overexpression in MHCC97H and Huh7 cells. (L) Western blotting analysis of PHLPP2 ubiquitination in HEK293T cells transfected with wild‐type FBXO32 or FBXO32‐G243R mutants. (M) Representative western blotting analysis showing K48‐linked PHLPP2 ubiquitination in HEK293T cells. (N) Representative western blotting analysis showing K48‐linked PHLPP2 ubiquitination at K592 and K942 sites in HEK293T cells. (O) Western blotting analysis of the relative PHLPP2 abundance and p‐AKT expression in control and FBXO32‐overexpressing HEK293T cells transfected with wild‐type PHLPP2 or PHLPP2‐K592R/K942R (K2R) mutants.

To further explain molecular mechanisms via which FBXO32 adjusts AKT signaling pathway, we presumed that E3 ligase FBXO32's ability of increasing AKT phosphorylation reflected decreased levels and/or activities of AKT phosphatases, like PHLPPs (PHLPP1 and PHLPP2), PP1, PP2A, and PTEN (Figure 5A). We surveyed variations in their levels and observed only PHLPP2 protein levels decreased in FBXO32‐overexpressing cells (Figure 5B). We performed further immunoprecipitation (IP) assays utilizing an anti‐Flag antibody in Flag‐FBXO32‐overexpressing cells to validate protein–protein interactions and confirmed that PHLPP2 exhibited a strong binding ability to FBXO32 (Figure 5C). This result was further corroborated by endogenous IP assays and molecular docking analyses (Figure 5D,E). Additionally, immunofluorescence experiments demonstrated FBXO32 and PHLPP2 colocalization (Figure 5F). Western blot analysis confirmed that FBXO32 negatively regulates PHLPP2 protein expression in HCC cells without affecting its mRNA levels (Figure 5G,H). Collectively, these findings suggest that FBXO32 interacts with and decreases the PHLPP2 protein levels.

To explore how FBXO32 regulates PHLPP2 expression, we treated cells using cycloheximide (CHX) to cut off protein synthesis and checked PHLPP2 protein levels. Our outcomes implied that FBXO32 promoted PHLPP2 degradation (Figure 5I). Furthermore, we treated FBXO32‐overexpressing and control cells with lysosome inhibitor chloroquine (CQ) or proteasome inhibitor MG132 and found that only MG132 blocked the FBXO32 overexpression‐induced decrease in PHLPP2 protein levels (Figure 5J). This demonstrated that PHLPP2 was degraded via the ubiquitin–proteasome pathway. FBXO32 knockdown and overexpression reduced and increased PHLPP2 ubiquitination, respectively (Figure 5K). Importantly, wild‐type FBXO32 significantly promoted PHLPP2 ubiquitination, unlike the inactive FBXO32‐G243R mutant (Figure 5L). Furthermore, K48‐linked ubiquitination is closely pertaining to ubiquitin–proteasome‐mediated degradation. Based on this, we hypothesized that FBXO32 promotes K48‐linked PHLPP2 ubiquitination (Figure 5M). We further predicted the ubiquitination sites of PHLPP2 using GPS‐Uber (Table S1) and confirmed that FBXO32 mediates its polyubiquitination through lysine residues K592 and K942 (Figure 5N). Additionally, K592 and K942 mutants exhibited a significant reduction in PHLPP2 degradation and AKT signaling activation upon FBXO32 overexpression (Figure 5O). Overall, these results exhibited that FBXO32 binds to PHLPP2 directly and propels its K48‐linked ubiquitination at the K592 and K942 sites.

### 2.6 FBXO32 Promotes HCC Progression via the PHLPP2–AKT Signaling Pathway

**FIGURE 6:**
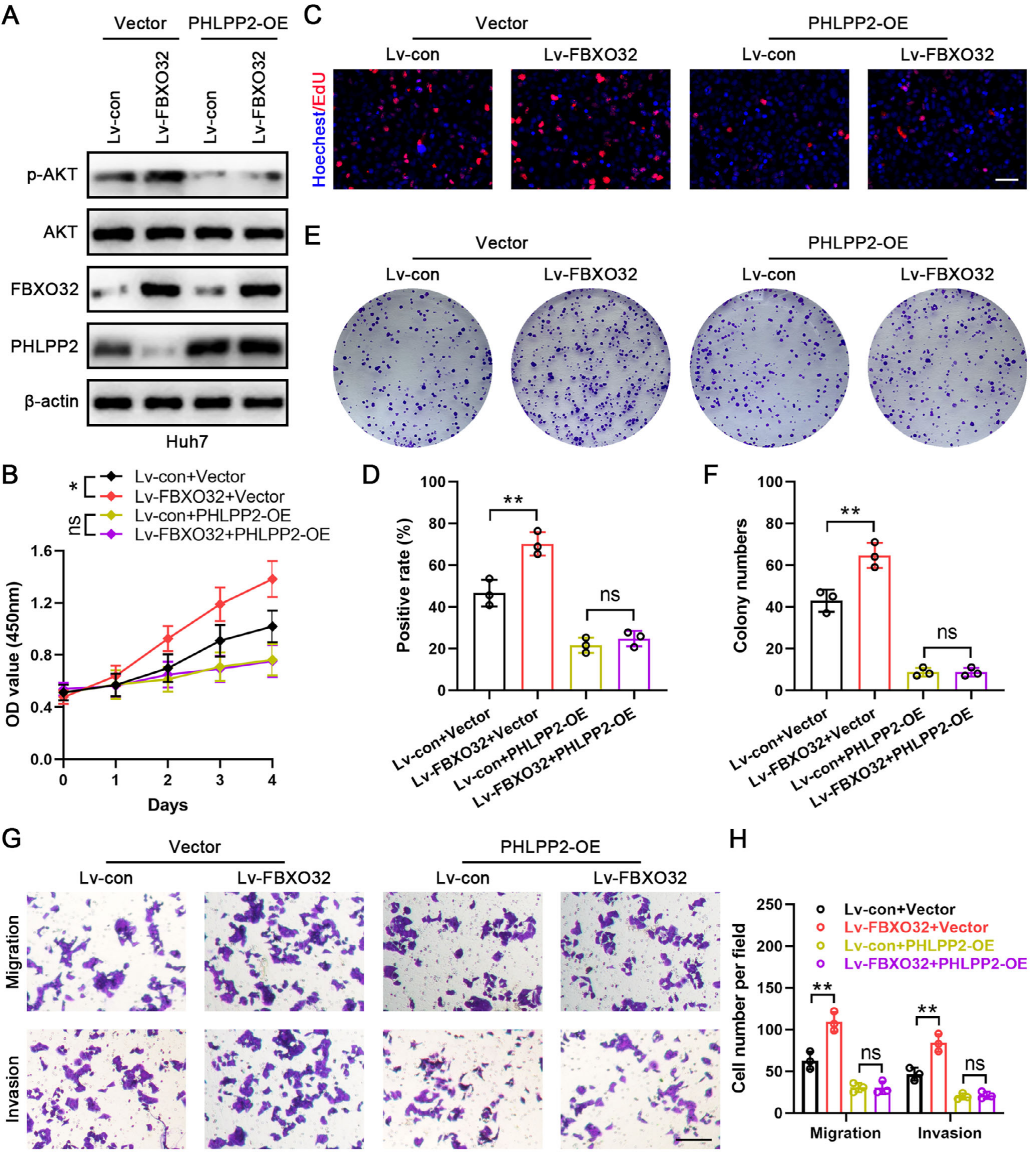
FBXO32 function is PHLPP2‐dependent. (A) Western blot analysis showing FBXO32, PHLPP2, total AKT, and phosphorylated AKT protein levels in the indicated Huh7 cells. (B) CCK‐8 assay results for Huh7 cells viability in the indicated groups (*n* = 3). (C, D) EdU assay results and proliferation efficiency of the indicated Huh7 cells (*n* = 3). Scale bar, 50 µm. (E, F) Colony formation assay results for Huh7 cells in the indicated groups (*n* = 3). (G, H) Representative transwell assay results and corresponding statistical analysis for migration and invasion of Huh7 cells in the indicated groups (*n* = 3). Scale bar, 50 µm. All data are presented as the means ± SD, *p < 0.05; **p < 0.01; ***p < 0.001; ns, not significant.

For assessing if FBXO32's effects upon HCC progression were dependent upon PHLPP2–AKT pathway, we infected Lv‐con and Lv‐FBXO32 Huh7 cells with a PHLPP2‐overexpressing (PHLPP2‐OE) lentivirus or a control lentivirus (vector). Western blot analysis validated that PHLPP2‐OE mitigated FBXO32‐induced AKT activation (Figure 6A). CCK‐8, EdU, and colony formation assays demonstrated that PHLPP2 overexpression abrogated FBXO32 upregulation‐induced increase in cell viability and proliferation efficiency (Figure 6B–F). Transwell assays revealed that PHLPP2 overexpression abrogated the FBXO32 upregulation‐induced enhancement of HCC cell migration and invasion (Figure 6G,H). Collectively, these data demonstrated that FBXO32 regulated HCC progression through the PHLPP2–AKT signaling pathway.

### 2.7 FBXO32 Upregulation Is Ascribed to DNA Promoter Hypomethylation

**FIGURE 7:**
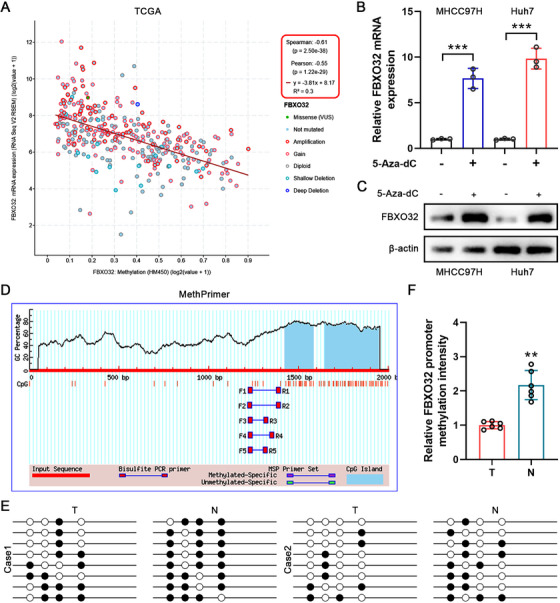
DNA hypomethylation upregulates FBXO32 in HCC. (A) The cBioPortal database was employed to analyze the association between DNA methylation levels and *FBXO32* mRNA expression in HCC. (B) *FBXO32* mRNA expression levels were evaluated in HCC cells after treatment with 5‐Aza‐dC (*n* = 3). (C) FBXO32 protein expression in MHCC97H and Huh7 cells post‐5‐Aza‐dC treatment. (D) Possible CpG islands in the FBXO32 promoter sequence screening using MethPrimer. (E, F) The DNA methylation status of FBXO32 CpG islands in randomly chosen HCC and matched adjacent normal tissues was determined by bisulfite sequencing PCR (BSP; *n* = 6). Unmethylated and methylated CpG sites are represented by open and filled circles, respectively. All data are presented as the means ± SD, *p < 0.05; **p < 0.01; ***p < 0.001; ns, not significant.

To further explore FBXO32 upregulation mechanism in HCC, bioinformatic analysis exhibited that *FBXO32* mRNA expression was pertaining to DNA methylation levels in a negative way (Figure 7A). We exposed MHCC97H and Huh7 cells that demonstrate relatively high and low FBXO32 expression, respectively, to 5‐Aza‐dC, a broadly applied DNA methyltransferase inhibitor. Both FBXO32 mRNA and protein expression were notably upregulated in 5‐Aza‐dC‐exposed cells than in untreated cells, meaning negative correlation of FBXO32 expression with DNA methylation (Figure 7B,C). Furthermore, bioinformatics analysis implied that FBXO32 promoter region contained typical CpG islands (Figure 7D). Further analysis revealed that the FBXO32 promoter was hypomethylated in tumor tissues, as confirmed by bisulfite sequencing PCR (BSP; Figure 7E,F). In summary, outcomes herein suggest that HCC's FBXO32 rise may be ascribed to DNA promoter hypomethylation. Upregulated FBXO32 promotes K48‐linked PHLPP2 polyubiquitination through K592 and K942 residues, inducing PHLPP2 degradation, activating PI3K–AKT signaling pathway, and consequently propelling malignant HCC progression.

### 2.8 Targeting FBXO32 Alleviates HCC Malignant Progression

**FIGURE 8:**
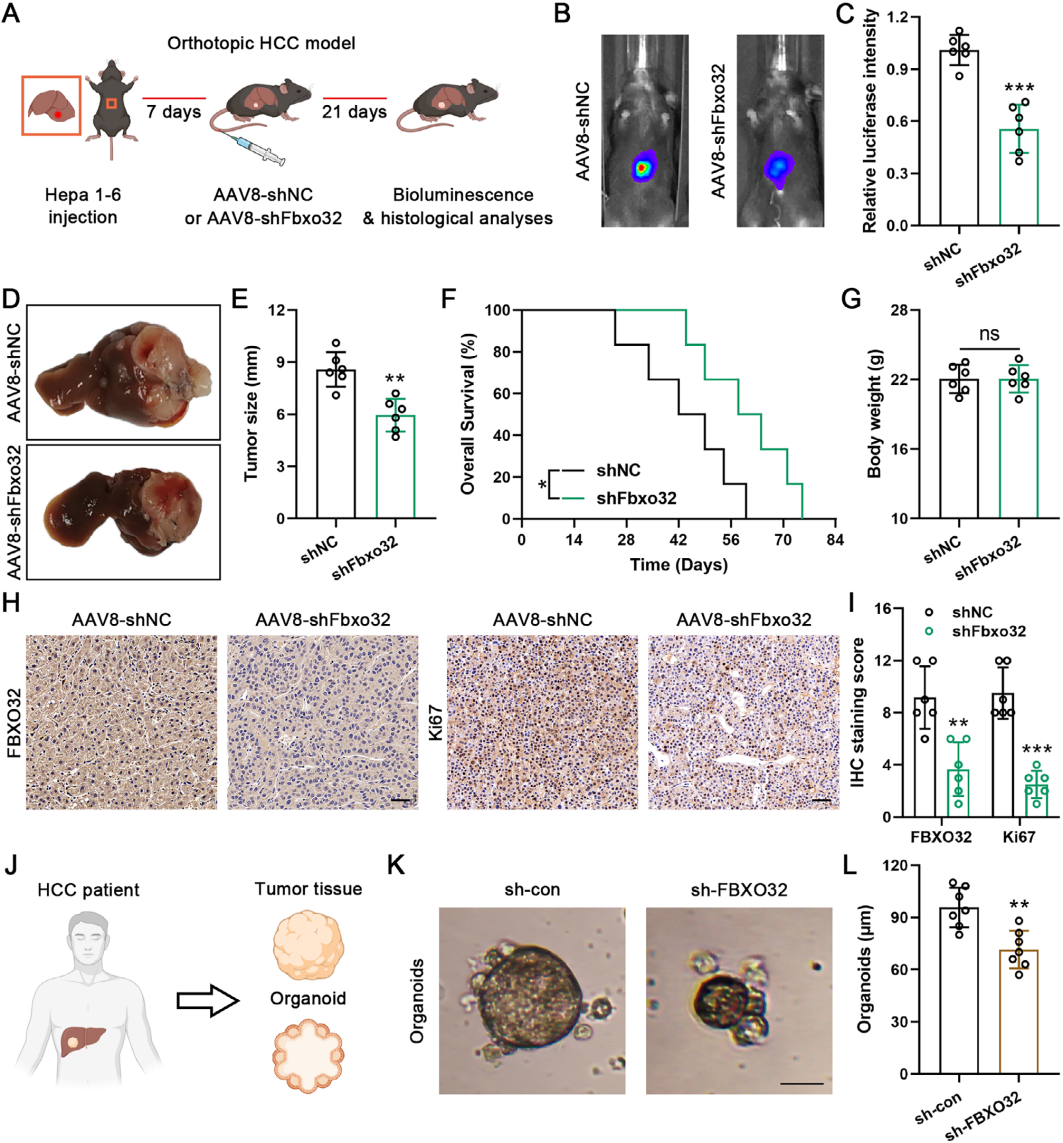
Targeting FBXO32 inhibits tumor growth. (A) Schematic representation of the treatment plan in the orthotopic tumor transplantation model, created using images from BioRender.com. (B) Bioluminescence imaging of orthotopic tumors (*n* = 6). (C) Luciferase intensity quantification in the indicated groups. (D) Representative images of liver orthotopic tumors. (E) Tumor size quantification in the indicated groups. (F) Survival curves from the orthotopic tumor model in the indicated groups. (G) Body weight of mice in the indicated groups. (H) Histological analysis of FBXO32‐ and Ki67‐stained orthotopic tumors. Scale bars, 50 mm. (I) Quantitative results of immunohistochemical (IHC) staining scores of tumor tissues in indicated groups. (J) Schematic diagram of human HCC tumor organoid establishment, created using images from BioRender.com. (K, L) Analysis of HCC organoid diameter (*n* = 7). Scale bars, 50 mm. All data are presented as the means ± SD, *p < 0.05; **p < 0.01; ***p < 0.001; ns, not significant.

We surveyed therapeutic potential of FBXO32 knockdown upon HCC progression utilizing an orthotopic mouse model (Figure 8A). Adeno‐associated virus (AAV) type 8 vector‐mediated RNA interference targeting Fbxo32 (AAV‐shFbxo32) dramatically suppressed orthotopic tumor growth derived from luciferase‐tagged mouse HCC cell line Hepa1‐6 in C57BL/6 mice, as evidenced by the reduced luciferase bioluminescence intensity (Figure 8B,C). Furthermore, infection with AAV‐shFbxo32 significantly decreased tumor size and notably extended the survival of mice without significantly changing body weight (Figure 8D–G). IHC analysis revealed a significant reduction in FBXO32 levels after AAV8‐shFbxo32 treatment, and Ki67 staining further demonstrated that targeting FBXO32 inhibited tumor growth (Figure 8H,I). For extending such outcomes to patient models, we applied a patient‐derived organoid (PDO) model (Figure 8J). Consistent with the observations in the mouse model, targeting FBXO32 in the PDO model significantly inhibited tumor growth (Figure 8K,L). To sum up, this work offers robust proof supporting therapeutic potential of hepatic FBXO32 knockdown for mitigating HCC growth. Such outcomes mean that targeting FBXO32 might act as alternative precise HCC therapy. However, further research should validate the clinical relevance of this approach.

## 3 Discussion

Patients with liver cancer typically face a poor prognosis, with many diagnosed at an unresectable stage [25]. Approximately 70% of those undergoing curative surgery eventually experience local or distant recurrence [26]. Hence, a comprehensive insight into intrinsic and extrinsic cellular mechanisms driving HCC progression is critical for developing effective therapies [27]. This study identifies FBXO32 as a novel HCC progression driver. We noticed that FBXO32 was upregulated in human HCC specimens and significantly correlated with advanced stages and poor prognosis. Targeting FBXO32 is a potential treatment strategy for inhibiting tumor progression in HCC. It means that FBXO32 can serve as an alternative biomarker for early detection and prognosis prediction and a target for novel therapeutic approaches. Further exploration of FBXO32‐targeting strategies may reduce tumor recurrence and improve survival outcomes in HCC patients.

DNA methylation refers to an epigenetic mechanism involving transfer of a methyl group to C5 position of cytosine, forming 5‐methylcytosine [28]. Such a modification moderates gene expression via enrolling proteins involved in gene repression or inhibiting binding of transcription factors to DNA [29]. In tumors, DNA hypomethylation is an ordinary epigenetic alteration that causes aberrant activation of oncogenes [30, 31]. In this study, we found that DNA hypomethylation may be crucial to regulating FBXO32 expression and propel HCC progression.

Ubiquitination regulation, one of the most vital post‐translational modifications, is crucial to tumorigenesis [32]. FBXO protein family is critical to ubiquitination process as E3 ligases, propelling tumor progression [33]. In particular, the ubiquitination regulation of FBXO32 is involved in various tumors through both oncogenic and tumor‐suppressive functions [34, 35]. Our work found that elevated FBXO32 levels promoted K48‐linked PHLPP2 polyubiquitination at K592 and K942 residues, leading to PHLPP2 degradation, PI3K–AKT signaling pathway activation, and subsequent malignant HCC progression.

PHLPP2, a phosphatase with PH domains, plays a central role in modulating AKT signaling by removing phosphate groups from serine residues, accordingly restraining AKT phosphorylation [36]. This regulation is crucial for maintaining proper cellular functions. For instance, miR‐25‐3p decreases PHLPP2 expression, accordingly activating PI3K–AKT–mTOR signaling pathway, which finally causes macrophage M2 polarization [37]. Additionally, the transcriptional repressor SNAI2 may promote glioma stem cell proliferation through AKT pathway activation via downregulating PHLPP2 [38]. Moreover, mutant p53 exerts its gain‐of‐function effect via transcriptionally restraining PHLPP2 and activating AKT, accordingly suppressing immune response and promotes tumor growth [39].

In addition to epigenetic modifications, PHLPP2 is also regulated by post‐translational ubiquitination. For example, TRIM22‐mediated PHLPP2 degradation causes AKT–p53–p21 signaling pathway activation, finally giving rise to cellular senescence [40]. Furthermore, TRIM46 amplification is associated with decreased PHLPP2 levels, increasing p‐AKT levels and promoting glycolysis in lung adenocarcinoma [41]. Similarly, USP46‐mediated PHLPP1 and PHLPP2 stabilization decreases cell proliferation and tumorigenesis in colon cancer cells [42]. In another context, KCTD17 binds to phosphorylated PHLPP2, targeting it for ubiquitin‐mediated degradation, thereby promoting hepatic steatosis [43]. Moreover, RNF149 confers cisplatin resistance in esophageal squamous cell carcinoma through PHLPP2 ubiquitination [44]. Similarly, MARCH1 promotes oral squamous cell carcinoma growth via PHLPP2 ubiquitination [45]. Collectively, such outcomes emphasize diverse roles of ubiquitination in adjusting PHLPP2, whereas underscore its critical impact on various aspects of cancer biology.

This study had some limitations. Most of our functional and mechanistic studies were based on HCC cell lines, which might not utterly capture diverse heterogeneity of human HCC. Such findings’ clinical significance requires further validation. Additionally, although our study primarily focused on HCC, aberrant FBXO32 expression has been observed in various malignant tumors. Consequently, pharmacological FBXO32 inhibition may improve tumor therapeutic responses in other cancer types, which warrants further investigation.

## 4 Conclusion

Our study displayed that FBXO32 interacts with PHLPP2 and propels its ubiquitination and degradation in HCC cells. These findings uncover a novel part of FBXO32 in HCC, implying that FBXO32 upregulation in HCC cells decreases PHLPP2 levels, further increasing p‐AKT levels and promoting tumor malignancy. Collectively, our data establish FBXO32 as an independent prognostic biomarker for HCC and promote its malignant progression. Furthermore, this study offers detailed mechanistic insights and multimodel experimental evidence of HCC progression, highlighting FBXO32 as a promising target for precise HCC treatment.

## 5 Materials and Methods

### 5.1 Tissues and Cell Lines

We acquired fifty pairs of HCC and adjacent normal tissues intraoperatively from patients who had not received antitumor therapy. The HCC and HEK293T cell lines adopted herein were gained from Cell Bank of Type Culture Collection in Shanghai, China and kept in Dulbecco's modified Eagle's medium (DMEM) supplemented with 10% fetal bovine serum (Gibco, NY, USA) under standard conditions at 37°C with 5% CO_2_.

### 5.2 RNA Extraction and Quantitative Reverse Transcription PCR

We extracted RNA from HCC tissues and cell lines utilizing TRIzol reagent (Invitrogen, USA). We reverse transcribed isolated RNA to generate complementary DNA (cDNA), which served as a template for quantitative reverse transcription PCR (qRT‐PCR) analysis. For normalizing *FBXO32* expression level, we utilized human β‐actin gene expression level as a reference (Table S2).

### 5.3 Western Blotting

Total proteins were extracted using RIPA buffer (Servicebio, Wuhan, China) on ice, and then separated by SDS‐PAGE (Servicebio) and transferred to PVDF. We incubated membranes utilizing corresponding primary antibodies at 4°C overnight, rinsed them three times with PBS, and cultured them for 2 h utilizing secondary antibodies. ECL signals were detected on PVDF membranes using the ECL Chemiluminescence kit (A and B, Servicebio), with β‐actin serving as a loading control.

### 5.4 Immunohistochemistry

We deparaffinized paraffin‐embedded tissues in xylene and rehydrated utilizing a graded ethanol series. We retrieved antigens using citrate buffer (pH 6.0) in a microwave oven. We blocked tissue slides utilizing hydrogen peroxide and incubated them via specific antibodies.

### 5.5 Cell Line Transfection

The concentrated virus was used to infect cells at 50% confluency in a 60 mm dish with 5 µg/mL polybrene. We selected infected cells using 2 µg/mL puromycin (Merck, Darmstadt, Germany) and performed plasmid transfection with Lipofectamine 3000 (Invitrogen) according to the manufacturer's instructions (Table S3).

### 5.6 Cell Proliferation Assays

For the CCK‐8 assay, HCC cells were cultured in 96‐well plates, treated with CCK‐8 solution, incubated at 37°C, and absorbance was measured at 450 nm. For colony formation assays, HCC cells were seeded in 6‐well plates, cultured for 10 days, fixed, stained with crystal violet, and counted. For the EdU assay, HCC cells were seeded in 12‐well plates, treated with 50 µM EdU, incubated at 37°C, fixed, permeabilized, and stained with Hoechst 33342. EdU‐positive cells were quantified in five randomly selected fields per well.

### 5.7 Wound Healing Assays

Cells were cultured in 6‐well plates, and a wound was created using a 200 µL pipette tip once 80% confluence was reached. Wound area was imaged at 0 and 24 h, and relative motility was calculated as [(s1 − s2)/s1].

### 5.8 Transwell Assays

Transwell assays were performed by seeding 1 × 10^4^ cells in serum‐free medium into the upper chamber and serum‐containing medium into the lower chamber. After 24 h, migrated cells were fixed, stained with crystal violet, and counted. For invasion assays, Matrigel (Corning) was precoated on the upper chamber.

### 5.9 In Vivo Mouse Model

To evaluate the in vivo effects of FBXO32 on HCC cell malignancy, 1 × 10^6^ transfected cells in PBS were injected subcutaneously into 4‐week‐old nude mice. Mice with similar body weights were randomly assigned to experimental and control groups, and tumor size was measured weekly. For lung metastasis assays, HCC cells stably expressing luciferase‐tagged sh‐con, sh‐FBXO32, Lv‐con, or Lv‐FBXO32 were used. Regarding to establishing metastatic tumor model, we injected PBS containing 1 × 10^6^ cells into tail veins of nude mice. We performed bioluminescent imaging (BLI) after 4 weeks for identifying metastases. For orthotopic HCC model, we inoculated 2 × 10^6^ Hepa1‐6 cells in 20 µL serum‐free DMEM/Matrigel (1:4) into liver of 6‐week‐old male C57BL/6 mice. After 7 days, 100 µL AAV8‐shNC or AAV8‐shFbxo32 (10^12^ pfu/mL) were delivered via tail vein injection. After 3 weeks, BLI analysis was performed to determine the tumor burden. The livers were harvested to examine the liver weight and tumor size and fixed for histological analyses.

### 5.10 PDO Preparation

Fresh HCC tumor tissues from patients were thoroughly washed three times with prechilled PBS containing penicillin–streptomycin. The tissues were immediately cut into 3–5 mm pieces and digested with 5 mM EDTA on ice for 1 h. The digestion was considered complete when cell aggregates were observed under a microscope. The digested tissue was mixed with Matrigel and carefully transferred to a 24‐well plate for further cultivation.

### 5.11 IP Assay

We transfected cells utilizing desired plasmid and lysed in ice‐cold IP buffer. Resulting cell lysates, containing target protein, were cultured overnight at 4°C utilizing demonstrated antibody‐conjugated beads. Following incubation, we eluted and analyzed bead‐bound proteins by western blotting utilizing appropriate primary and secondary antibodies.

### 5.12 Ubiquitination Assay

We transfected cells utilizing desired plasmid and lysed using lysis buffer with 10% SDS. Lysates were denatured via heating at 100°C for 10 min. We subsequently diluted supernatant 10‐fold with lysis buffer. After that, we gathered indicated molecules using IP as previously described.

### 5.13 BSP Analysis

The extracted genomic DNA was treated with bisulfite utilizing a Methylation‐Gold kit according to manufacturer's instructions. Regarding to quantitative methylation analysis, methPrimer online bioinformatics tool was used to identify potential CpG islands in FBXO32 and design specific primers. Bisulfite‐treated genomic DNA was amplified using BSP primers (Table S4).

### 5.14 Quantification and Statistical Analysis

Publicly available RNA‐seq data and clinical information for HCC patients were obtained from the TCGA database (Level 3 open‐access data, downloaded in 2021). Gene expression analysis and survival correlation studies were conducted using the UALCAN platform, which integrates TCGA Level 3 data for open‐access analysis (Table S5). We performed all statistical analyses utilizing GraphPad Prism software and IBM SPSS Statistics (Table S6). Student's *t*‐test and one‐way ANOVA before Tukey's post hoc test were used to measure significance levels. We denote results as mean ± SD from at least three independent tests. We denoted significance levels as below: *p < 0.05, **p < 0.01, ***p < 0.001; ns, not significant.

## Author Contributions

Jingjing Dai and Jiali Xu conceived and designed the study, performed experiments, wrote the original draft, and provided funding support and supervision. Shu Chen, Kai Yu, Zhengming Deng, and Xiaopei Hao analyzed data, drew the schematic diagram, and conducted bioinformatics analysis. Ping Shi and Zhengzheng Wang provided expert consultation. All the authors have read and approved the final manuscript.

## Ethics Statement

Our work was approved by institutional ethics board of First Affiliated Hospital of Nanjing Medical University (approval number: IACUC2304040). We informed patients or their relatives of proper use of human specimens and all of them signed informed consent forms.

## Conflicts of Interest

The authors declare no conflicts of interest.

## Supporting information




**Supporting Table 1**: PHLPP2 ubiquitination site prediction.
**Supporting Table 2**: qRT‐PCR primers used in this study.
**Supporting Table 3**: The target sequences of lentiviral shRNAs used in this study.
**Supporting Table 4**: Primers for BSP.
**Supporting Table 5**: List of online databases used in this study.
**Supporting Table 6**: List of Softwares.

## Data Availability

All data supporting these outcomes are available upon request.
