# Supplementary material for: Hypomethylation‐Enhanced F‐Box Protein 32 Promotes Hepatocellular Carcinogenesis via Ubiquitin‐Mediated PHLPP2 Degradation
Source: MedComm (2020). 2025 Sep 23;6(10):e70410. doi: 10.1002/mco2.70410 (PMC12457717; doi:10.1002/mco2.70410)
Supplement: Supplementary file 1 — Supporting Table 1: PHLPP2 ubiquitination site prediction. Supporting Table 2: qRT‐PCR primers used in this study. Supporting Table 3: The target sequences of lentiviral shRNAs used in this study. Supporting Table 4: Primers for BSP. Supporting Table 5: List of online databases used in this study. Supporting Table 6: List of Softwares. [file MCO2-6-e70410-s001.docx]

**Hypomethylation-enhanced F-Box Protein 32 promotes hepatocellular carcinogenesis via ubiquitin-mediated PHLPP2 degradation**

Running title: F-Box Protein 32 Promotes HCC Progression

Shu Chen^1,#,*.^ Kai Yu^2,#.^ Zhengming Deng^3,#.^ Xiaopei Hao^4,#.^ Ping Shi^5.^ Zhengzheng Wang^4,*.^ Jiali Xu^6,*.^ Jingjing Dai^5,*.^

^1^Department of Hepatobiliary Pancreatic Spleen Surgery, The Affiliated Hospital of Jiangsu University, Zhenjiang 212001, China.

^2^Hepatobiliary Center, The First Affiliated Hospital with Nanjing Medical University; Key Laboratory of Liver Transplantation, Chinese Academy of Medical Sciences; NHC Key laboratory of Hepatobiliary cancers, Nanjing, Jiangsu Province, China.

^3^Department of General Surgery, Jiangsu Province Hospital of Chinese Medicine, Affiliated Hospital of Nanjing University of Chinese Medicine, Nanjing 210029, China.

^4^Department of Hepatobiliopancreatic Surgery, The Affiliated Cancer Hospital of Zhengzhou University & Henan Cancer Hospital, Zhengzhou, 450008, China.

^5^Department of Infectious Diseases, The First Affiliated Hospital with Nanjing Medical University, Nanjing, Jiangsu Province 210029, China.

^6^Department of Anesthesiology, Jinling Hospital, Affiliated Hospital of Medical School, Nanjing University, Nanjing, Jiangsu Province, China.

^#^These authors contributed equally.

| ^*^Correspondence authors: | Jingjing Dai, email: daijingjing@njmu.edu.cn |
| --- | --- |
|  | Jiali Xu, email: xjl15950466318@163.com |
|  | Zhengzheng Wang, email: wangzz818@126.com |
|  | Shu Chen, email: chenshuzj@163.com |

**Supplementary Tables**

| **Supplementary table 1**. PHLPP2 ubiquitination site prediction. | | |
| --- | --- | --- |
| Position | Code | Score |
| 592 | K | 0.7524 |
| 942 | K | 0.6589 |
| 245 | K | 0.5088 |
| 1011 | K | 0.4960 |
| 934 | K | 0.4954 |
| 916 | K | 0.4386 |
| 681 | K | 0.4355 |

| **Supplementary table 2**. qRT-PCR primers used in this study. | |
| --- | --- |
| Primer name | Sequence |
| β‐actin F | GCTCGTCGTCGACAACGGCTC |
| β‐actin R | CAAACATGATCTGGGTCATCTTCTC |
| FBXO32 F | TGAGCGACCTCAGCAGTTAC |
| FBXO32 R | GCGCTCCTTCGTACTTCCTT |

| **Supplementary table 3**. The target sequences of lentiviral shRNAs used in this study. | |
| --- | --- |
| Lentiviral shRNA | Target sequence |
| sh1 | CAACAAGGAGGTATACAAT |
| sh2 | GGAAGAAGATGTATTTCAAAC |
| sh3 | CCAAGGAAAGAGCAGTATGGA |
| sh-Fbxo32 | CCTATGAAGATGCCACACAAT |

| **Supplementary table 4**. Primers for BSP. | |
| --- | --- |
| Primer name | Sequence |
| FBXO32 F | TTGGCCCTGGGTACATCATTTAACC |
| FBXO32 R | GACTGGTTAGTGACAGCTAAGGGGC |

| **Supplementary table 5**. List of online databases used in this study. | |
| --- | --- |
| Database | Online link |
| GPS-Uber | https://gpsuber.biocuckoo.cn/ |
| UALCAN database | https://ualcan.path.uab.edu/ |
| GEPIA database | <http://gepia.cancer-pku.cn/> |
| Gene set enrichment analysis (GSEA) | <https://www.broadlnstitute.org/gsea/> |
| STRING database | <https://string-db.org/> |
| DAVID database | <https://david.ncifcrf.gov/> |
| MethPrimer | https://www.urogene.org/ |
| cBioPortal database | https://www.cbioportal.org/ |

| **Supplementary table 6**. List of Softwares. | |
| --- | --- |
| Software | version |
| GraphPad Prism | 8.0 |
| ImageJ | Open source |
| IBM SPSS statistics | 26.0 |
| Pymol | 3.11 |
| Origin | 9.8 |
| Quantification tool for Methylation Analysis | Open source |
